# Internal disinhibition predicts 5‐year weight regain in the National Weight Control Registry (NWCR)

**DOI:** 10.1002/osp4.22

**Published:** 2016-01-18

**Authors:** J. Lillis, J. G. Thomas, H. Niemeier, R. R. Wing

**Affiliations:** ^1^ Department of Psychiatry and Human Behavior Warren Alpert Medical School of Brown University, The Miriam Hospital/Weight Control and Diabetes Research Center Providence RI USA; ^2^ Department of Psychology University of Wisconsin‐Whitewater Whitewater WI USA

**Keywords:** Coping, disinhibition, obesity, weight loss

## Abstract

**Background:**

Maintenance of weight loss remains elusive for most individuals. One potential innovative target is internal disinhibition (ID) or the tendency to eat in response to negative thoughts, feelings or physical sensations. Individuals high on ID do worse on average in standard behavioural treatment programmes, and recent studies suggest that disinhibition could play a significant role in weight regain.

**Purpose:**

The purpose of the current study was to examine whether ID was associated with weight change over 5 years of follow‐up in the National Weight Control Registry, a registry of individuals who have successfully lost weight and maintained it.

**Methods:**

From the National Weight Control Registry, 5,320 participants were examined across 5 years. Weight data were gathered annually. The disinhibition subscale of the Eating Inventory was used to calculate internal disinhibition and External Disinhibition (ED) and was collected at baseline, year 1, year 3 and year 5. Linear mixed models were used to estimate the weight loss maintained across follow‐up years 1 to 5 using ID and ED as baseline and prospective predictors.

**Results:**

Internal disinhibition predicted weight regain in all analyses. ED interacted with ID, such that individuals who were high on ID showed greater weight regain if they were also higher on ED.

**Conclusions:**

The ID scale could be a useful screening measure for risk of weight regain, given its brevity. Improved psychological coping could be a useful target for maintenance or booster interventions.

## Introduction

Obesity remains a significant public health problem. Although clinically significant weight loss is achievable, maintaining the weight loss is challenging 1. Thus, it is important to identify behavioural factors associated with poor maintenance that could lead to treatment innovations designed to improve weight loss maintenance.

Disinhibited eating or more simply ‘disinhibition’, defined as the tendency to impulsively or opportunistically eat, has been implicated as a key factor in poor weight control given its association with body mass index (BMI) 2, overeating 3, 4 and weight cycling 5. However, findings regarding the role of disinhibition as a predictor of weight loss and weight loss maintenance have been inconsistent, resulting in confusion regarding the importance of the construct 6, 7, 8. Some of the confusion can be attributed to the use of several different measures of disinhibition, although the results are variable even within the most common measure, the disinhibition subscale of the Eating Inventory (EI; aka. Three‐factor Eating Questionnaire 9).

Niemeier and colleagues 10 conducted a new factor analysis of the EI disinhibition scale using two samples of individuals who had participated in longitudinal weight loss research studies (total *N* = 3,631) and found that disinhibition, as measured by the EI, is better represented by two factors: internal disinhibition (ID), which is the tendency to eat in response to cognitive or emotional cues, and external disinhibition (ED), which is the tendency to eat in response to environmental cues 10. In recent studies, both lower baseline levels of ID 10 and a greater decrease in ID early in weight loss treatment 11 predicted better weight loss outcomes at 18 and 12 months, respectively, whereas ED was not related to weight loss outcomes. These findings may help to explain the inconsistent pattern of results obtained when the broader disinhibition construct is studied, which combines the effects of ID and ED.

Disinhibition sometimes remains high after weight loss treatment 8, which could create a vulnerability to weight regain. Recent studies further suggest that disinhibition could play a more significant role in weight regain, as opposed to initial weight loss 5. Thus, it is important to study disinhibition and its association with weight change over time among individuals who have been successful at initial weight loss.

The National Weight Control Registry (NWCR) 12 is a large ongoing study of individuals who maintained a weight loss of at least 13.6 kg (30 lb) for at least 1 year. Participants are followed annually after enrolling in the NWCR, making it an ideal context in which to study associations between disinhibition (both ID and ED) and weight change over time. Only one prior study has examined the role of internal disinhibition in weight loss maintenance, but in this study, also conducted with NWCR participants, follow‐up was limited to 1 year. Baseline ID, but not ED, was predictive of weight change at 1‐year follow‐up 10.

The purpose of the current study was to examine whether ID and/or ED were associated with the weight loss maintained over the 5‐year follow‐up period. We hypothesize that ID, but not ED, will be associated with poorer weight outcomes across 5 years. If eating in response to internal or external cues plays a role in weight regain, it could be targeted with innovative treatment approaches.

## Methods and procedures

### Participants

Eligibility criteria for the NWCR 12 include ≥30 lb (13.6 kg) weight loss, maintained for ≥1 year. Although there are currently over 10,000 NWCR members, we focused on 5,400 individuals who lost weight without bariatric surgery and who have reached the 5‐year follow‐up. Participants were also required to have completed the EI and reported their weight at enrollment and at least one of the five annual follow‐up questionnaires. The final sample was 5,320 participants.

### Procedures

Participants are recruited through unpaid coverage in various media (e.g. newspapers, magazines and television) and join by calling a 1‐800 number or visiting the NWCR website 12. Participants are asked to verify their weight loss by providing physician documentation, before and after photos, or a collateral testimony from family or friends. Questionnaires are then sent at entry and once annually for 5 years. Participants may optionally re‐consent to providing another 5 years of self‐reported weight change, but the EI is not administered beyond year 5.

### Measures

#### Demographics and weight history

Demographic data on age, sex and race/ethnicity, as well as weight history data including lifetime maximum weight and duration of weight maintenance, were collected via self‐report at baseline. At each follow‐up assessment, participants report their current weight and height. The reliability and validity of self‐reported weights of registry members have been demonstrated 13. The outcome measure, percent weight loss, was calculated as follows: ([self‐reported current weight − self‐reported lifetime highest weight] ÷ self‐reported lifetime highest weight).

#### Disinhibition

The EI 9 was administered at baseline and 1‐, 3‐ and 5‐year follow‐up. This 51‐item measure has been used extensively in studies of weight control and has well‐established psychometric properties that have been reviewed elsewhere 14. As in previous studies 10, 11, ID and ED were computed using eight and six non‐overlapping items on the disinhibition subscale of the EI, respectively. As mentioned earlier, ID measures eating in response to internal experiences, such as thoughts or feelings. For example, one item reads ‘When I feel lonely, I console myself by eating’. ED measures eating in response to exposure to food or food‐related stimuli. For example, one item reads, ‘When I am with someone who is overeating, I usually overeat too’. A psychometric analysis based on factor analytic techniques was used to identify the ID and ED subscales, which have proved valid in reliable in subsequent studies 10, 11.

### Statistical analysis

Analyses were conducted using PASW Statistics 19 (SPSS Inc., 2009, Chicago, IL, USA www.spss.com). Linear mixed models using a maximum likelihood approach were used to estimate percent weight loss across follow‐up years 1 to 5. Unconditional models were used to examine percent weight loss in the absence of covariates and evaluate the variance components associated with subject intercepts and slopes for the time variable (years since baseline), which were both found to vary by individual and were therefore treated as random effects in all models.

In a first ‘baseline predictors’ analysis, baseline ID and ED values were used to predict percent weight loss across 5 years of follow‐up. In a second ‘prospective predictors’ analysis, ID and ED at baseline and follow‐up years 1 and 3 were used to predict percent weight loss at follow‐up years 1, 2 and 4, respectively (i.e. the year after ID and ED were assessed), thereby demonstrating whether a change in ID and/or ED was associated with a prospective change in body weight.

For both analyses, ID and ED were entered as simultaneous predictors of percent weight loss to determine the effect of each type of disinhibition when the other was controlled. In a second step, ID and ED were allowed to interact to predict weight loss. Participant age, gender, ethnicity (non‐Hispanic Caucasians versus all others), weight loss upon NWCR enrollment (highest ever weight − weight at enrollment) and number of years of weight loss maintenance prior to enrollment were entered as covariates in all analyses. Tests of significance were conducted at *α* = 0.05.

Our analytic approach allowed data from all participants with at least one follow‐up assessment to contribute to the ‘baseline predictors’ analysis, thereby limiting the impact of missing data 15. In order to be included in the ‘prospective predictors’ analysis, participants were required to have complete data from at least one of the consecutive‐years pairs (i.e. baseline and 1 year, 1 and 2 years, or 3 and 4 years). The number of participants contributing to each analysis is reported in the subsequent discussion. The baseline demographic characteristics and weight history of participants who did versus did not complete the 5‐year assessment were compared using *t*‐test and chi‐square, as appropriate.

## Results

### Participant characteristics

The 5,320 participants had a mean ± standard deviation (SD) BMI of 25.07 ± 4.5 at time of entry to the NWCR. The sample was 75% female, 95% Caucasian and highly educated (85% had attended college and 60% had at least a bachelor's‐level college degree). The mean ± SD age for the sample was 47.0 ± 12.2 years old. Compared with those that did not complete the 5‐year assessment (*n* = 2,741, 51.6%), participants who completed the 5‐year assessment (*n* = 2,574, 48.4%) were slightly older (48.7 ± 11.9 versus 45.4 ± 12.43 years old, *p* < 0.001) and had a slightly lower BMI at enrollment (24.8 ± 4.1 versus 25.3 ± 4.8, *p* < 0.001), weight loss at enrollment (29.6% ± 8.8% versus 30.8% ± 9.5% weight loss, *p* < 0.001) and duration of weight loss maintenance at enrollment (5.9 ± 7.6 versus 4.6 ± 6.2 years, *p* < 0.001). The 5‐year completers were also less likely to be African–American (1.4% versus 2.5%, *p* < 0.001) or Hispanic (0.7% versus 1.8%, *p* < 0.001) and were more likely to have at least some college education (85.7% versus 84.2% *p* < 0.001). There was no difference between 5‐year completers and non‐completers on gender (*p* = 0.546).

### Follow‐up completion rates

Participants completed 65% of the five yearly follow‐up assessments. The proportion of individuals completing a full assessment including weight and EI at each year of follow‐up was as follows: year 1 = 84%; year 2 = 77%; year 3 = 68%; year 4 = 50%; and year 5 = 48%. The ‘baseline predictors’ analysis included *n* = 5,320 participants, and the ‘prospective predictors’ analysis included 91.2% (*n* = 4,853) of those participants.

### Five‐year percent weight loss and disinhibition

Baseline and follow‐up values for percent weight loss, ID and ED are reported in Table 1. Participants entered the registry with a mean ± SD percent weight loss of 30.2 ± 9.2 (32.7 ± 16.8 kg) and having kept it off 5.2 ± 6.9 years. On average, participants were estimated to gain approximately 1.6 kg per year (*p* < 0.001). Small increases in ID and ED were also observed across the follow‐up period (*p's* < 0.001).

**Table 1:** Weight loss and disinhibition (internal and external) across 5 years of follow‐up

	Baseline (*n* = 5,320)	1‐year (*n* = 4,448)	2‐year (*n* = 4,076)	3‐year (*n* = 3,638)	4‐year (*n* = 2,673)	5‐year (*n* = 2,577)
Percent weight loss	30.2 ± 9.2	28.2 ± 10.0	26.5 ± 10.5	25.4 ± 10.9	24.3 ± 11.4	23.6 ± 11.5
Internal disinhibition	2.7 ± 2.2	2.8 ± 2.4	—	2.9 ± 2.5	—	3.1 ± 2.4
External disinhibition	2.4 ± 1.7	2.4 ± 0.1.7	—	2.5 ± 1.7	—	2.6 ± 1.7

### Baseline predictors analysis

Percent weight loss across the 5‐year follow‐up period was lower in the presence of higher baseline scores on both ID (coefficient = −0.36, SE = 0.05, *t* = 7.132, *p* < 0.001) and ED (coefficient = −0.26, SE = 0.06, *t* = 4.105, *p* < 0.001), but only baseline ID was significantly related to more rapid deterioration in percent weight loss (i.e. faster weight regain) across the 5‐year follow‐up (coefficient = −0.10, SE = 0.02, *t* = 4.781, *p* < 0.001). Baseline ED was not significantly associated with the rate of change in percent weight loss over 5 years (coefficient = 0.02, SE = 0.03, *t* = 0.676, *p* = 0.499). The interaction of baseline ID and ED was not statistically significant (coefficient = −0.02, SE = 0.01, *t* = 1.882, *p* = 0.060).

### Prospective predictors analysis

**Figure 1:**
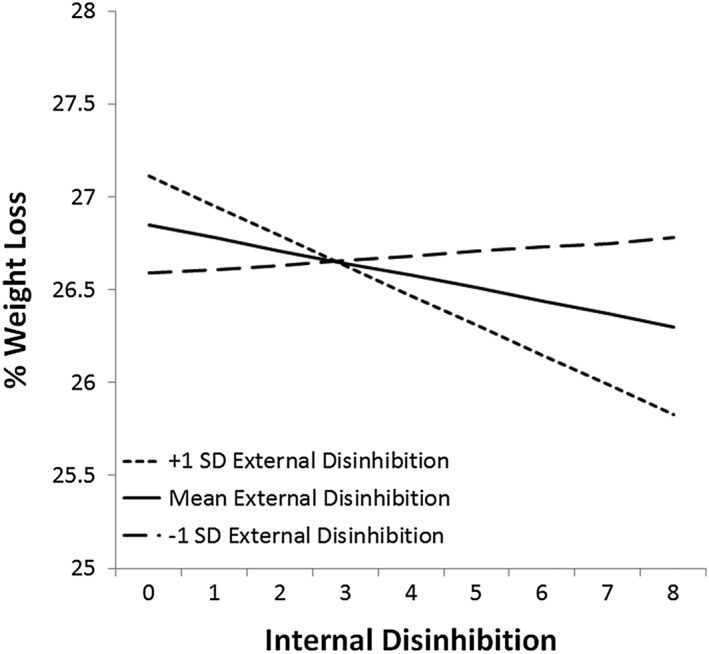
Weight change as a function of the interaction between internal and external disinhibition.

Internal disinhibition and ED scores at baseline and 1‐ and 3‐year follow‐up were used to predict weight change at 1‐, 2‐ and 4‐year follow‐up, respectively. When entered into the model simultaneously with baseline ID and ED values controlled, higher scores on ID (coefficient = −0.27, SE = 0.06, *t* = 2.963, *p* = 0.003) were significantly associated with lower percent weight loss (i.e. greater weight gain) at the following year's assessment. Scores on ED were not predictive of weight change (coefficient = −0.03, SE = 0.05, *t* = 0.741, *p* = 0.459). When ID and ED were allowed to interact, there was a statistically significant interaction effect (coefficient = −0.05, SE = 0.01, *t* = 3.842, *p* < 0.001) in which the trend towards greater regain at higher levels of ID was more pronounced at higher levels of ED (Figure 1).

## Discussion

The purpose of the current study was to examine whether ID was associated with weight change over 5 years of follow‐up in the NWCR, a registry of individuals who have successfully lost weight and maintained it. Consistent with our hypothesis, higher ID scores at baseline and 1‐ and 3‐year follow‐up were significantly associated with poorer weight loss and thus faster weight regain, at years 2, 4 and 5 after joining the NWCR. These results are consistent with, and extend on, a previous study showing that ID predicts poor response to weight loss treatment as well as weight regain over 1 year in the NWCR 10. Also consistent with our hypothesis, ED was not an independent predictor of weight regain, when controlling for ID. Interestingly, however, ED did interact with ID in the prospective analysis, such that individuals who were high on ID showed greater weight regain if they were also higher on ED. Although we did not make any specific hypothesis about possible interactions, this seems to indicate that ED may play some role in poor weight outcomes when high ID is present.

The ID scale could be a useful screening measure for risk of weight regain, given its brevity. The IE is already widely used in both research and clinical practice, making this potentially easy to implement. Individuals higher on ID could be screened for additional or more targeted interventions.

Internal disinhibition could be a useful target for maintenance or booster interventions that focus on improved psychological coping. For example, acceptance and mindfulness‐based techniques aim to reduce unhealthy forms of coping in relation to unwanted thoughts, feelings and bodily sensations 16. Given that ID indicates increased eating in response to internal cues, acceptance and mindfulness methods seems well situated to potentially improve outcomes. These techniques have been tested with promise in smaller weight loss intervention studies with positive outcomes 17, 18.

Further investigation is needed to explore the role of ED in weight regain. However if high ED leads to poorer outcomes in the presence of high ID, then treatments might need to target both internal and external cues when addressing triggers for disinhibition. Future studies should attempt to clarify if there are cutoffs or ‘types’ that are clinically meaningful in order to help clarify when it might be useful to target ID only or a combination of ID and ED.

This study has important limitations. A significant number of participants were lost by, or had not yet reached, 5‐year follow‐up. In addition, the NWCR contains only individuals who lost >30 lb and maintained the loss for >1 year. This will naturally limit the sample and could preclude the generalizability of the findings. In addition, the sample was composed of 95% Caucasian individuals, limiting the generalizability to diverse racial and ethnic groups.

The current study showed that ID could be an important predictor of weight regain and could prove valuable in screening for individuals who might benefit from additional intervention.

## Conflicts of Interest Statement

No conflict of interest was declared.

## References

[1] Barte JCM , ter Bogt NCW , Bogers RP , *et al.* Maintenance of weight loss after lifestyle interventions for overweight and obesity, a systematic review. Obes Rev 2010; 11: 899‐906. 20345430 10.1111/j.1467-789X.2010.00740.x

[2] Dykes J , Brunner EJ , Martikainen PT , Wardle J . Socioeconomic gradient in body size and obesity among women: the role of dietary restraint, disinhibition and hunger in the Whitehall II study. Int J Obes (Lond) 2004; 28: 262‐268. 10.1038/sj.ijo.0802523 14647173

[3] Van Strien T , Cleven A , Schippers G . Restraint, tendency toward overeating and ice cream consumption. Int J Eat Disord 2000; 28: 333‐338. 10942920 10.1002/1098-108x(200011)28:3<333::aid-eat11>3.0.co;2-#

[4] Westenhoefer J , Broeckmann P , Munch AK , Pudel V . Cognitive control of eating behavior and the disinhibition effect. Appetite 1994; 23: 27‐41. 7826055 10.1006/appe.1994.1032

[5] Bryant EJ , King NA , Blundell JE . Disinhibition: its effects on appetite and weight regulation. Obes Rev 2008; 9: 409‐419. 18179615 10.1111/j.1467-789X.2007.00426.x

[6] Foster GD , Wadden TA , Swain RM , *et al.* The Eating Inventory in obese women: clinical correlates and relationship to weight loss. Int J Obes (Lond) 1998; 22: 778‐785. 10.1038/sj.ijo.0800659 9725638

[7] Karlsson J , Hallgren P , Kral J , *et al.* Predictors and effects of long‐term dieting on mental well‐being and weight‐loss in obese women. Appetite 1994; 23: 15‐26. 7826054 10.1006/appe.1994.1031

[8] Cuntz U , Leibbrand R , Ehrig C , Shaw R , Fichter MM . Predictors of post‐treatment weight reduction after in‐patient behavioral therapy. Int J Obes (Lond) 2001; 25: S99‐S101. 10.1038/sj.ijo.0801710 11466600

[9] Stunkard AJ , Messick S . The 3‐factor Eating Questionnaire to measure dietary restraint, disinhibition and hunger. J Psychosom Res 1985; 29: 71‐83. 3981480 10.1016/0022-3999(85)90010-8

[10] Niemeier HM , Phelan S , Fava JL , Wing RR . Internal disinhibition predicts weight regain following weight loss and weight loss maintenance. Obesity 2007; 15: 2485‐2494. 17925475 10.1038/oby.2007.295

[11] Butryn ML , Thomas JG , Lowe MR . Reductions in internal disinhibition during weight loss predict better weight loss maintenance. Obesity 2009; 17: 1101‐1103. 19180064 10.1038/oby.2008.646 PMC5524144

[12] Klem ML , Wing RR , McGuire MT , Seagle HM , Hill JO . A descriptive study of individuals successful at long‐term maintenance of substantial weight loss. Am J Clin Nutr 1997; 66: 239‐246. 9250100 10.1093/ajcn/66.2.239

[13] McGuire MT , Wing RR , Klem ML , Lang W , Hill JO . What predicts weight regain in a group of successful weight losers? J Consult Clin Psychol 1999; 67: 177‐185. 10224727 10.1037//0022-006x.67.2.177

[14] Karlsson J , Persson LO , Sjostrom L , Sullivan M . Psychometric properties and factor structure of the Three‐Factor Eating Questionnaire (TFEQ) in obese men and women. Results from the Swedish Obese Subjects (SOS) study. Int J Obes (Lond) 2000; 24: 1715‐1725. 10.1038/sj.ijo.0801442 11126230

[15] Schafer JL , Graham JW . Missing data: our view of the state of the art. Psychol Methods 2002; 7: 147‐177. 12090408

[16] Hayes SC , Luoma JB , Bond FW , Masuda A , Lillis J . Acceptance and commitment therapy: model, processes and outcomes. Behav Res Ther 2006; 44: 1‐25. 16300724 10.1016/j.brat.2005.06.006

[17] Lillis J , Hayes SC , Bunting K , Masuda A . Teaching acceptance and mindfulness to improve the lives of the obese: a preliminary test of a theoretical model. Ann Behav Med 2009; 37: 58‐69. 19252962 10.1007/s12160-009-9083-x

[18] Forman EM , Butryn ML , Hoffman KL , Herbert JD . An open trial of an acceptance‐based behavioral intervention for weight loss. Cogn Behav Pract 2009; 16: 223‐235.

